# Psychological and personality factors in type 2 diabetes mellitus, presenting the rationale and exploratory results from The Maastricht Study, a population-based cohort study

**DOI:** 10.1186/s12888-016-0722-z

**Published:** 2016-01-27

**Authors:** Fleur E. P. van Dooren, Johan Denollet, Frans R. J. Verhey, Coen D. A. Stehouwer, Simone J. S. Sep, Ronald M. A. Henry, Stef P. J. Kremers, Pieter C. Dagnelie, Nicolaas C. Schaper, Carla J. H. van der Kallen, Annemarie Koster, Frans Pouwer, Miranda T. Schram

**Affiliations:** Department of Internal Medicine, Maastricht University Medical Centre, Randwycksingel 35, 6229 EG Maastricht, The Netherlands; CARIM Cardiovascular Research Institute Maastricht, Maastricht University, Maastricht, The Netherlands; Department of Medical and Clinical Psychology, CoRPS – Center of Research on Psychology in Somatic diseases, Tilburg University, Tilburg, The Netherlands; MHeNS – Alzheimer Centre Limburg, School for Mental Health and Neuroscience, Maastricht University, Maastricht, The Netherlands; Department of Social Medicine, Maastricht University, Maastricht, The Netherlands; CAPHRI School for Public Health and Primary Care, Maastricht University, Maastricht, The Netherlands; Department of Epidemiology, Maastricht University, Maastricht, The Netherlands; Department of Health Promotion, NUTRIM School for Nutrition, Toxicology and Metabolism, Maastricht University Medical Centre+, Maastricht, The Netherlands

**Keywords:** Type 2 diabetes, Cohort, Design, Exploratory results, Depression, Anxiety, Personality

## Abstract

**Background:**

Strong longitudinal evidence exists that psychological distress is associated with a high morbidity and mortality risk in type 2 diabetes. Little is known about the biological and behavioral mechanisms that may explain this association. Moreover, the role of personality traits in these associations is still unclear. In this paper, we first describe the design of the psychological part of The Maastricht Study that aims to elucidate these mechanisms. Next, we present exploratory results on the prevalence of depression, anxiety and personality traits in type 2 diabetes. Finally, we briefly discuss the importance of these findings for clinical research and practice.

**Methods:**

We measured psychological distress and depression using the MINI diagnostic interview, the PHQ-9 and GAD-7 questionnaires in the first 864 participants of The Maastricht Study, a large, population-based cohort study. Personality traits were measured by the DS14 and Big Five personality questionnaires. Type 2 diabetes was assessed by an oral glucose tolerance test. Logistic regression analyses were used to estimate the associations of depression, anxiety and personality with type 2 diabetes, adjusted for age, sex and education level.

**Results:**

Individuals with type 2 diabetes had higher levels of depressive and anxiety symptoms, odds ratios (95 % CI) were 3.15 (1.49; 6.67), 1.73 (0.83–3.60), 1.50 (0.72–3.12), for PHQ-9 ≥ 10, current depressive disorder and GAD-7 ≥ 10, respectively. Type D personality, social inhibition and negative affectivity were more prevalent in type 2 diabetes, odds ratios were 1.95 (1.23–3.10), 1.35 (0.93–1.94) and 1.70 (1.14–2.51), respectively. Individuals with type 2 diabetes were less extraverted, less conscientious, less agreeable and less emotionally stable, and similar in openness to individuals without type 2 diabetes, although effect sizes were small.

**Conclusions:**

Individuals with type 2 diabetes experience more psychological distress and have different personality traits compared to individuals without type 2 diabetes. Future longitudinal analyses within The Maastricht Study will increase our understanding of biological and behavioral mechanisms that link psychological distress to morbidity and mortality in type 2 diabetes.

## Background

Type 2 diabetes mellitus is an increasingly common chronic disease, afflicting an estimated 171 million individuals with type 2 diabetes in 2000 to 366 million in 2030 [1]. Individuals with type 2 diabetes are at higher risk to develop micro- and macrovascular complications and have higher mortality rates [2, 3]. Depression is a frequent co-morbid condition in individuals with type 2 diabetes. Two meta-analyses report that depression is almost twice as common in type 2 diabetes compared to individuals without diabetes [4, 5]. In addition, depressive symptoms appeared to be highly persistent and/or recurrent in type 2 diabetes. For example, Nefs et al. [6] showed that 66 % of individuals with type 2 diabetes who had a high depression score, still had a high score two to three years later. Furthermore, multiple studies have linked depression to a variety of adverse health outcomes among individuals with type 2 diabetes, like a reduced quality of life [7], less optimal self-care behaviors and higher HbA_1c_ levels [8], a higher risk of micro- and macrovascular complications [9], higher mortality rates [10] and increased health care costs [11]. However, the majority of these studies had a cross-sectional design, thereby limiting the possibility to assess the temporal sequence of the observed associations.

In contrast, little is known about the risk of other forms of distress, for example those that are related to anxiety. Anxiety frequently co-occurs with depressive symptoms [12, 13], and therefore appears to be common in type 2 diabetes. Similar to depression, anxiety disorders typically have a chronic and recurrent life course, and elevated anxiety symptoms and diagnosed anxiety disorders in type 2 diabetes have been associated with reduced quality of life [14], poor glycemic control [15, 16] and diabetes complications [17]. Yet, the association between type 2 diabetes and anxiety has not been studied extensively. The mechanisms underlying this association may show similarities with those of depression as anxiety is associated with deregulation of the hypothalamic-pituitary-adrenal (HPA) axis [18], but also life-style factors such as dietary behavior, physical inactivity and obesity may be relevant [19].

In addition to psychological variables, personality factors emerge as potential vulnerability factor for adverse health outcomes. Type D personality refers to the combination of negative affectivity (tendency to experience negative emotions) and social inhibition (tendency to inhibit self-expression) [20]. The Type D or ‘distressed’ personality has been related to poor health status, morbidity and mortality in cardiovascular disease [21–23], although negative findings have also been reported [24, 25]. One potential mechanism through which Type D may exert a negative influence on health is suboptimal self-care behavior. Also, biological mechanisms like a dysfunctional HPA-axis, increased heart rate and blood pressure have been proposed [26–29]. Nonetheless, to our knowledge few studies have explored the potential behavioral and biological mechanisms that may link Type D with adverse health outcomes in individuals with type 2 diabetes [30].

Prospective detailed longitudinal studies combining extensive phenotyping on depression, anxiety and type 2 diabetes with a focus on a broad range of determinants are needed to elucidate the complex underlying pathophysiology between depression, type 2 diabetes and adverse health outcomes. The Maastricht Study has been designed as a large, prospective population-based cohort study that fits these needs. The niche of this study lies in the advanced assessment of both depression and type 2 diabetes, but also in the vast number of participants of the study, as we aim to include 10,000 participants with substantial oversampling of individuals with type 2 diabetes. To our knowledge, The Maastricht Study is the first attempt to address the different mechanisms underlying the complex association of depression and type 2 diabetes comprehensively in one population.

The main aim of the current paper is to describe the psychological design and research questions within the framework of The Maastricht Study, which amongst others, focuses on psychological variables and personality in the context of type 2 diabetes. In addition, we also present the findings of psychological and personality assessments in the first 862 participants of The Maastricht Study to document the feasibility of these assessments in this study. We will conclude with theories on possible mechanisms linking psychological distress with type 2 diabetes, and with suggestions for future research.

## Methods

### The Maastricht Study

#### Study design, subject eligibility and recruitment

The Maastricht Study is an observational prospective population-based cohort study, enriched with type 2 diabetes individuals. The rationale and methodology have been described previously [31]. In brief, the study focuses on the etiology, pathophysiology, complications and comorbidities of type 2 diabetes and is characterized by an extensive phenotyping approach. The study population will be enriched with type 2 diabetes participants for reasons of efficiency; i.e., to increase the statistical power to identify any potential contrasts between individuals with and without type 2 diabetes.

Eligible for participation were all individuals aged between 40 and 75 years and living in the southern part of the Netherlands. Participants were recruited through mass media campaigns and from the municipal registries and the regional Diabetes Patient Registry via mailings. The regional Diabetes Patient Registry is kept by the regional association of General Practitioners and the Maastricht University Medical Centre. This registry includes individuals that apply to their general practitioner with health complaints which lead to the diagnosis of type 2 diabetes, and individuals that are diagnosed with type 2 diabetes after cardiovascular screening. The registry virtually includes all individuals with type 2 diabetes in primary, secondary or tertiary medical care in the “Maastricht and Heuvelland” region.

Recruitment was stratified according to known type 2 diabetes status for reasons of efficiency. The present report includes cross-sectional data from the first 866 participants, who completed the baseline survey between November 2010 and March 2012. The examinations of each participant were performed and completed within a time window of 3 months. The study has been approved by the institutional medical ethical committee (Medisch Ethische Commissie aZM/UM, (NL31329.068.10) and the Netherlands Health Council under the Dutch “Law for Population Studies” (Permit 131088-105234-PG). All participants gave written informed consent.

#### Research questions

To further increase our understanding of the association between psychosocial variables and adverse health outcomes in type 2 diabetes, we aim to address the following research questions within the framework of The Maastricht Study:

1. What is the effect of type 2 diabetes on the development and recurrence of depression?
2. What is the effect of depression on the development and progression of type 2 diabetes? In particular impaired glucose metabolism, micro- and macrovascular complications, mortality and, quality of life will be evaluated.
3. What mechanisms may explain these associations, with a specific focus on psychosocial (e.g. burden of disease, anxiety or personality traits), biological (e.g. vascular factors, inflammation, hyperglycemia, deregulated HPA-axis) and behavioral (e.g. physical inactivity, poor diet, smoking) mechanisms?
4. What is the potential added value (additional variance explained), of anxiety and personality in the association of psychological variables with type 2 diabetes?

In this paper we did not address these research questions, as we present a descriptive study with exploratory first results of The Maastricht Study.

#### Diagnosis of diabetes

To determine glucose metabolism, all participants (except those who use insulin) underwent a standardized 7-point oral glucose tolerance test (OGTT) after an overnight fast. Blood samples are taken at baseline, and 15, 30, 45, 60, 90 and 120 min after ingestion of a 75 g glucose drink. For safety reasons, participants with a fasting glucose level above 11.0 mmol/l, as determined by a finger prick, did not undergo the OGTT. Glucose metabolism is defined according to the WHO 2006 criteria into normal glucose tolerance (NGT), impaired fasting glucose (IFG), impaired glucose tolerance (IGT) and type 2 diabetes [32]. These criteria maintain the following thresholds; for NGT fasting plasma glucose level of ≤ 6.0 mmol/L, for IFG fasting plasma glucose level of 6.1–6.9 mmol/L and a 2-h plasma glucose of < 7.8 mmol/L, for IGT fasting plasma glucose of <7.0 mmol/L and a 2-h plasma glucose of ≥ 7.8 and ≤ 11.1 mmol/L, for type 2 diabetes fasting plasma glucose of ≥ 7.0 mmol/L or a 2-h plasma glucose of ≥ 11.1 mmol/L. Individuals that used glucose-lowering medication were classified as having type 2 diabetes. Individuals with type 1 diabetes (*n* = 4), were excluded from the analyses.

#### Diagnosis of depression and anxiety disorder

The Mini-International Neuropsychiatric Interview (MINI) [33] was used to determine whether participants suffered from an anxiety disorder or a depressive disorder. The MINI is a short diagnostic structured interview, used to assess the presence of minor or major depressive disorder, or an anxiety disorder in the preceding 2 weeks and in the past (lifetime depression) according to the DSM-IV. The MINI includes questions about age at onset of first depressive episode and number of episodes, and was administered by four trained investigators. The following DMS-IV classifications were distinguished: depressive disorder (major and minor depression), lifetime (recurrent) depressive episode and dysthymia. Participants were diagnosed as having a depressive disorder if they had at least one core symptom (i.e. depressed mood or loss of interest) and at least one other symptom of depression (minor depression), or at least one core symptom (i.e. depressed mood or loss of interest) and at least four other symptoms of depression (major depression in DSM-IV). If a person suffers from two or more of these episodes in his or her life, a diagnosis of lifetime (recurrent) depression was made. Dysthymia represents a longer-lasting but symptomatically milder disorder, defined by at least two years of continued mood disturbance, along with at least two associated symptoms.

The MINI was also used to assess the presence of panic disorder, agoraphobia and social phobia, according to the DSM-IV criteria. With regard to panic disorder assessed by means of the MINI, four screening questions were applied to rule out the diagnosis when answered negatively. With use of decision tree logic, positive responses to screening questions are explored by further investigation of other diagnostic criteria. Participants were diagnosed with panic disorder if they answered all screening questions positively, and had at least four symptoms of panic disorder. For a diagnosis of agoraphobia and social phobia all screening questions had to be answered affirmatively.

#### Symptoms of depression and anxiety

Depressive symptoms were assessed by a validated Dutch version of the 9-item Patient Health Questionnaire (PHQ-9) [34]. The PHQ-9 is a self-administered questionnaire based on the DMS-IV (Diagnostic and Statistical Manual of Mental Disorders, Fourth Edition) [35] criteria for a major depressive disorder. It comprises nine items rated on a 4-point scale, ranging from 0 = “not at all” to 3 = “nearly every day”. Response options can generate a continuous score ranging from 0 (no symptoms) to 27 (all symptoms present nearly every day); scores 10–14 represent moderate and 15–27 moderately severe to severe depression symptoms. The PHQ-9 scale was also used as a dichotomous variable with a pre-defined cut-off level of 10, which represents the presence of clinically relevant depressive symptoms.

Anxiety symptoms were assessed by a validated Dutch version of the 7-item Generalized Anxiety Disorder scale (GAD-7) [36]. The GAD-7 is a self-administered questionnaire based on the DMS-IV criteria for a generalized anxiety disorder (GAD). It comprises seven items rated on a 4-point scale, ranging from 0 = “not at all” to 3 = “nearly every day”. Total scores range from 0 to 21, with higher scores indicating the presence of more (severe) anxiety symptoms. The GAD-7 can be used both as a continuous score, with minimal (0–4), mild (5–9), moderate (10–14) and severe (15–21) symptoms, as well as a dichotomous variable, with a pre-defined cut-off of 10, which represents a probable diagnosis of GAD. Both questionnaires have been used in other studies on diabetes [37–40].

#### Personality traits

Personality traits were assessed by a validated Dutch version of the Type D Scale-14 (DS14) [20] and the Quick Big Five [41]. The DS14 assesses Type D personality, which is characterized by negative affectivity, the tendency to experience negative emotions across time/situations, and social inhibition, the tendency to inhibit the expression of emotions in social interactions to avoid disapproval. The DS14 comprises 14 items, which are scored on a 5-point rating scale ranging from 0 = “false” to 4 = “true”, and uses two subscales, measuring level of negative affectivity and social inhibition. Total scores on both subscales range from 0 to 28. The DS-14 is used as a dichotomous score, with subjects obtaining a score of ≥ 10 on both subscales are considered to have a Type D personality. Previously, the DS14 has also been validated in primary care patients with type 2 diabetes [30].

The Quick Big Five is a Dutch, shortened version of the Big Five Factor Structure developed by Goldberg et al. [42]. The Quick Big Five measures five main personality domains; extraversion, agreeableness, conscientiousness, emotional stability and openness to experience, with 6-items assigned for each domain. This questionnaire consists of 30 items, which are scored on a 7-point rating scale ranging from 1 = “very inaccurate” to 7 = “very accurate”. Total scores for each personality trait range from 6 to 42. All main personality traits are named according to the ‘high scoring’ end of each scale. Higher scores on each trait indicate a strong presence of this personality trait and affiliated behaviors, and will be less able to sustain the tendencies of the low scorer, and vice versa. Both questionnaires on personality have been used in previous research on diabetes [30, 43].

#### Sociodemographic and clinical characteristics

As previously described [31, 44] diabetes duration, partner status, socioeconomic status based on education level and smoking status were assessed by self-report questionnaire. Educational level was assessed during a cognitive assessment interview and was classified into eight categories commonly used in The Netherlands [45]: 1) no education; 2) primary education; 3) lower vocational education; 4) intermediate general secondary education; 5) intermediate vocational education; 6) higher general secondary education; 7) higher vocational education; and 8) university. For this study, three groups were created for educational level: low (levels 1–3), middle (levels 4–6) and high (levels 7 and 8).

Smoking behavior was based on self-report of smoking cigarettes, cigars and/or pipe tobacco and divided into three categories, i.e. non-smoker, former smoker and current smoker. Additionally, lifetime smoking was expressed as pack-years; one pack-year was defined as one packet (=20 cigarettes) per day, smoked over a course of 1 year. Fasting venous blood samples were used to assess glucose levels, HbA_1c_ and lipid profile. Medication use was assessed by interview. Height and weight were measured to calculate body mass index. Office blood pressure was measured three times after 10 min of seated rest, and the mean of these three measurements was used for analyses.

#### Statistical analyses

Statistical analyses were performed with SPSS 20 for Windows. Four participants with type 1 diabetes were excluded from the analyses. Comparisons of sociodemograpic and clinical characteristics, depression, anxiety and personality traits between individuals with and without type 2 diabetes were made by use of independent sample t-tests or Chi-square tests. We used logistic regression analyses to estimate the associations of depression, anxiety and personality traits with type 2 diabetes (outcome variable) adjusted for age, sex and education level.

## Results

### Sociodemographic and clinical characteristics

Sociodemographic and clinical characteristics of the first 862 participants of The Maastricht Study are presented in Table 1 according to type 2 diabetes status. Individuals with type 2 diabetes were older, more frequently male and had a lower educational level as compared to individuals without type 2 diabetes. As expected, HbA1c levels were higher in type 2 diabetes, as were body mass index, systolic and diastolic blood pressure as compared to individuals without type 2 diabetes. Individuals with type 2 diabetes had a favorable lipid profile, which is probably due to the high frequency of lipid-lowering medication use in type 2 diabetes.

**Table 1:** Sociodemographic and clinical characteristics of The Maastricht Study participants

	No type 2 diabetes (*n* = 609)	Type 2 diabetes (*n* = 253)	*p*-value
Age, years	58 ± 8.6	64 ± 7.0	< 0.001
Male sex, n (%)	296 (49 %)	176 (70 %)	< 0.001
Partner, n (%)	515 (85 %)	208 (85 %)	0.98
Educational level, % low/middle/high	12/39/49	28/47/25	<0.001
HbA1c, %	5.65 ± 0.37	6.91 ± 0.88	< 0.001
Diabetes duration, years	-	7 [3–11]	
Diabetes medication, % no/oral/insulin	-	22/57/21	
Smoking, % never/former/current	34/49/17	22/64/14	
Pack years	4 [0–18]	15 [0–33]	< 0.001
Body mass index, kg/m^2^	26.3 ± 4.0	29.8 ± 4.7	< 0.001
Systolic blood pressure, mmHg	133 ± 18	146 ± 19	< 0.001
Diastolic blood pressure, mmHg	76 ± 10	78 ± 10	0.004
Total cholesterol, mmol/l	5.5 ± 1.1	4.4 ± 1.0	< 0.001
HDL cholesterol, mmol/l	1.40 ± 0.41	1.12 ± 0.40	< 0.001
LDL cholesterol, mmol/l	3.5 ± 1.0	2.6 ± 0.9	< 0.001
Triglycerides, mmol/l	1.3 ± 0.9	1.8 ± 1.0	< 0.001

Data are presented as mean ± standard deviation (SD), number (percentage) or as median [inter quartile range (IQR)], unless otherwise indicated

Data on depression, anxiety and personality traits were available in *n* = 852 for the MINI interview, *n* = 757 for PHQ-9, *n* = 721 for GAD-7, *n* = 712 for type D personality, *n* = 712 for Big Five personality traits. Missing data were mainly due to not completing the questionnaires.

### Prevalence of psychological distress and personality traits in type 2 diabetes

Table 2 shows the prevalence of depressive and anxiety symptoms and personality traits in relation to type 2 diabetes. A statistically significantly higher level of depressive symptoms was found in type 2 diabetes as measured by the PHQ-9. This difference was less pronounced for depressive disorder assessed by the MINI, and absent for lifetime depressive episode. Dysthymia was more prevalent in type 2 diabetes, though the numbers in both groups were small. Anxiety symptoms were not significantly different in individuals with and without type 2 diabetes. Anxiety disorders as assessed by the MINI had a low prevalence, therefore, formal testing of differences was only possible for agoraphobia. Current agoraphobia was somewhat more prevalent in individuals with type 2 diabetes, while the prevalence of panic disorder and social phobia were similar between groups. Type D personality was statistically significantly associated with type 2 diabetes, as were its two constituting components ‘social inhibition’ and ‘negative affectivity’. The Big Five showed statistically significantly lower levels in type 2 diabetes for the personality traits ‘extraversion’, ‘agreeableness’, ‘conscientiousness’ and ‘emotional stability’, yet ‘openness to experience’ was not different between individuals with and without type 2 diabetes.

**Table 2:** Prevalence of depressive and anxiety symptoms and personality traits in individuals with/ without type 2 diabetes

	No type 2 diabetes	Type 2 diabetes	*p*-value
Depressive symptoms			
PHQ-9 score	**2.46 ± 3.18**	**3.26 ± 4.53**	**0.020**
PHQ-9 ≥ 10	23 (4.2 %)	16 (7.8 %)	0.081
Depressive disorder			
Depressive disorder (MINI)	33 (5.5 %)	22 (8.8 %)	0.105
Lifetime depressive episode (MINI)	220 (36.5 %)	86 (34.3 %)	0.538
Dysthymia (MINI)	5 (0.8 %)	7 (2.8 %)	^a^
Anxiety symptoms			
GAD-7 score	2.59 ± 3.59	2.83 ± 4.01	0.444
GAD-7 ≥ 10	29 (5.4 %)	14 (7 %)	0.424
Anxiety disorders			
Current panic disorder	4 (0.7 %)	3 (1.2 %)	^a^
Lifetime panic disorder	26 (4.3 %)	6 (2.4 %)	^a^
Current agoraphobia	35 (5.8 %)	24 (9.6 %)	0.073
Current social phobia	9 (1.5 %)	3 (1.2 %)	^a^
Type D personality			
Social inhibition	**8.55 ± 4.63**	**9.62 ± 5.27**	**0.013**
Social inhibition score ≥ 10	**189 (36.4 %)**	**87 (45.1 %)**	**0.038**
Negative affectivity	**7.47 ± 4.93**	**8.54 ± 5.65**	**0.020**
Negative affectivity score ≥ 10	**140 (27 %)**	**70 (36.3 %)**	**0.020**
Type D personality	**79 (15.2 %)**	**44 (22.8 %)**	**0.027**
Big Five personality traits			
Extraversion	**30.4 ± 6.5**	**28.9 ± 7.4**	**0.016**
Agreeableness	**34.8 ± 3.7**	**33.7 ± 4.8**	**0.004**
Conscientiousness	**32.6 ± 5.7**	**31.1 ± 5.9**	**0.002**
Emotional stability	**30.6 ± 6.3**	**29.5 ± 7.0**	**0.058**
Openness	**28.5 ± 6.5**	**28.0 ± 6.4**	**0.287**

Data are presented as mean ± standard deviation or number (percentage)

^a^ Formal testing was not possible due low numbers

Bold data reflect significant findings

### Association of type 2 diabetes with psychological distress and personality traits

In Table 3, logistic regression analyses adjusted for age, sex and education level showed a 10 % higher odds of having type 2 diabetes per point increase in PHQ-9 score, while a PHQ-9 level ≥10 was associated with a 3.15 times higher odds. Depressive disorder was not statistically significant associated with type 2 diabetes. With regard to anxiety, logistic regression analyses adjusted for age, sex and education level showed no statistically significant association with type 2 diabetes. For Type D personality, logistic regression analyses showed a 95 % higher odds for type 2 diabetes, while a slightly reduced odds of type 2 diabetes ranging between 3 and 6 % was observed for the various personality traits of the Big Five.

**Table 3:** The association of depression and anxiety symptoms and personality traits with type 2 diabetes, adjusted for age, sex and education level

	Type 2 diabetes versus no diabetes		
	Odds ratio^a^	(95 % CI)	*p*-value
Depressive symptoms			
PHQ-9 score	**1.10**	**(1.05–1.15)**	**<0.001**
PHQ-9 ≥ 10	**3.15**	**(1.49–6.67)**	**0.003**
Depressive disorder			
Depressive disorder (MINI)	1.73	(0.83–3.60)	0.156
Lifetime depressive episode (MINI)	0.97	(0.69–1.36)	0.862
Anxiety symptoms			
GAD-7 score	1.03	(0.99–1.08)	0.162
GAD-7 ≥ 10	1.50	(0.72–3.12)	0.280
Type D personality			
Social inhibition	**1.04**	**(1.00–1.08)**	**0.032**
Social inhibition score ≥ 10	1.35	(0.93–1.94)	0.112
Negative affectivity	**1.05**	**(1.02–1.09)**	**0.004**
Negative affectivity score ≥ 10	**1.70**	**(1.14–2.51)**	**0.009**
Type D personality	**1.95**	**(1.23–3.10)**	**0.005**
Big Five personality traits			
Extraversion	**0.97**	**(0.95–1.00)**	**0.023**
Agreeableness	**0.94**	**(0.90–0.98)**	**0.003**
Conscientiousness	**0.94**	**(0.91–0.97)**	**<0.001**
Emotional stability	**0.97**	**(0.94–0.99)**	**0.013**
Openness	1.00	(0.97–1.03)	0.938

^a^ Logistic regression analyses were adjusted for age, sex and education level

Bold data reflect significant findings

## Discussion

Our first exploratory results indicate a higher prevalence of both depressive and anxiety symptoms in type 2 diabetes. This is in line with previous studies [46, 47]. A similar trend was observed for depressive disorder, with a higher prevalence in type 2 diabetes, although the difference was not statistically significant. In addition, Type D personality was more prevalent in type 2 diabetes, while personality traits like extraversion, agreeableness, consciousness and emotional stability were slightly less prevalent.

There are a number of possible mechanisms that may explain the link between type 2 diabetes and psychological distress. Below, we will discuss a number of these mechanisms, with a particular focus on depression.

### Psychosocial mechanisms

The “psychological burden hypothesis” states that the burden of knowing that one has diabetes, the management of having a chronic illness, or having complications to cope with, contribute to higher levels of depression [48]. In line with this hypothesis, a meta-analysis showed that the occurrence of depression was particularly increased in known type 2 diabetes, but not in persons with screen-detected type 2 diabetes (who were unaware that they had diabetes), or those with impaired glucose metabolism [49]. Type 2 diabetes self-management is known to be burdensome, as it requires discipline and perseverance to adapt every day activities (diet, exercise, rest) to medication use and glucose levels [50]. Insulin therapy, particularly, may be associated with increased psychological distress [51]. Moreover, diabetes complications can cause functional limitations (reduced mobility, visual impairments, fatigue, pain) and a reduced quality of life [52]. In the Longitudinal Aging Study Amsterdam (LASA), depressive symptoms were particularly common in individuals with type 2 diabetes and comorbid diseases, and not in individuals with type 2 diabetes alone [53].

### Biological mechanisms

Several biological mechanisms have been suggested to underlie the association between psychological distress and type 2 diabetes. First, the vascular depression hypothesis may be especially relevant in type 2 diabetes, since diabetes is accompanied by abundant (cardio-)vascular disease. This hypothesis postulates that cerebral small vessel lesions can lead to depressive symptoms via damage to deep and frontal brain structures that are involved in mood regulation [54, 55]. Only few longitudinal studies evaluated the association between early vascular changes and depression, and found a positive association between markers of cerebral small vessel disease and levels of depressive symptoms and/or recurrent depression [56–58]. In addition, higher levels of arterial stiffening, an early phenomenon in the development of cardiovascular disease which is accelerated in type 2 diabetes, were found to be slightly higher in individuals with depression [59, 60]. Second, systemic low-grade inflammation is a key phenomenon in the development of cardiovascular disease [61], a common complication of type 2 diabetes, and also plays a role in the etiology of depression [62]. The inflammatory pathway is likely to involve endothelial dysfunction and oxidative stress, and markers of these processes have been related to depression [63–66] and are known risk markers for type 2 diabetes [67, 68]. However, studies that specifically focus on this topic in relation to depression and type 2 diabetes remain scarce [69]. Third, blood glucose levels themselves may be a compelling regulator for mood states as well. In particular, prolonged hyperglycemia or hypoglycemia are able to induce negative emotional states in type 2 diabetes [70]. The brain is particularly vulnerable to fluctuations in plasma glucose levels, since neurons do not possess an active glucose transporter. This means that high plasma glucose levels have a direct effect on intraneuronal glucose levels, which may result in the induction of oxidative stress via the polyol pathway, enhanced formation of advanced glycation endproducts (AGEs) and subsequent neuronal damage [71–73].

### Lifestyle and medication use

Lifestyle factors, such as obesity, physical inactivity, smoking, alcohol abuse and poor diet are well known risk factors for the development of type 2 diabetes [50, 74]. At the same time lifestyle improvements, including weight reduction and increased physical activity, are important aspects of diabetes management [75]. Most lifestyle factors are related to depression as well, which warrants their evaluation when examining the association between depression and adverse health outcomes in type 2 diabetes.

The long-term use of anti-depressants may provide a potential mechanism in the development of type 2 diabetes. A meta-analysis of both cross-sectional and longitudinal studies in depressive patients without diabetes showed a 50 % increased risk of type 2 diabetes after long-term use of antidepressants, which was not fully explained by weight gain associated with anti-depressant use [76].

### Limitations

Due to the cross-sectional design of this study, it is not possible to draw any conclusion regarding causality. Carefully analyzing longitudinal data is the best alternative, and The Maastricht Study aims to fulfill this need in the near future. We were not able to demonstrate a significant differences in the prevalence of depressive disorder between individuals with or without type 2 diabetes, while a difference was observed for depressive symptoms. We expect that this is due to a relatively low power, caused by the relatively low number of cases in the diabetes group. In addition, due to the limited number of participants and the descriptive and exploratory nature of this study, we did not control our analyses for multiple covariates. While the inclusion of this study is ongoing, we expect to be able to present fully adjusted analyses in a larger study sample in due time.

### Future research

The potential explanations for the association between psychological distress, type 2 diabetes and adverse health outcomes described here are not exclusive; the relationship is likely to be multifactorial. Clearly, the psychosocial, biological and lifestyle mechanisms that may explain the high co-occurrence of type 2 diabetes and psychological distress needs further research. Moreover, most of the above mentioned studies were based on cross-sectional data and conclude that longitudinal studies are needed to truly test the various hypotheses, as we intend to do within The Maastricht Study.

Within the framework of The Maastricht Study, a well-defined large and innovative population-based cohort study characterized by deep phenotyping, we aim to improve our knowledge on these complex associations. In specific, we aim to address the role of glucose metabolism and micro- and macrovascular complications on the development and recurrence of depression and vice versa. Furthermore, we aim to investigate the biological and behavioral mechanisms that may underlie these associations. Finally, the potential added value of anxiety and personality in the association of psychological variables with type 2 diabetes needs further study.

## Conclusions

Our first exploratory results of The Maastricht Study show a high prevalence of depression, anxiety and Type D personality in type 2 diabetes, supporting the clinical importance of the research aims within The Maastricht Study.
